# Anti-Diarrheal Mechanism of the Traditional Remedy Uzara via Reduction of Active Chloride Secretion

**DOI:** 10.1371/journal.pone.0018107

**Published:** 2011-03-30

**Authors:** Jörg D. Schulzke, Susanne Andres, Maren Amasheh, Anja Fromm, Dorothee Günzel

**Affiliations:** 1 Department of Gastroenterology, Section of General Medicine and Nutrition, Charité, Campus Benjamin Franklin, Freie Universität Berlin and Humboldt Universität zu Berlin, Berlin, Germany; 2 Institute of Clinical Physiology, Charité, Campus Benjamin Franklin, Freie Universität Berlin and Humboldt Universität zu Berlin, Berlin, Germany; Ulm University, Germany

## Abstract

**Background and Purpose:**

The root extract of the African Uzara plant is used in traditional medicine as anti-diarrheal drug. It is known to act via inhibition of intestinal motility, but malabsorptive or antisecretory mechanisms are unknown yet.

**Experimental Approach:**

HT-29/B6 cells and human colonic biopsies were studied in Ussing experiments *in vitro*. Uzara was tested on basal as well as on forskolin- or cholera toxin-induced Cl^−^ secretion by measuring short-circuit current (I_SC_) and tracer fluxes of ^22^Na^+^ and ^36^Cl^−^. Para- and transcellular resistances were determined by two-path impedance spectroscopy. Enzymatic activity of the Na^+^/K^+^-ATPase and intracellular cAMP levels (ELISA) were measured.

**Key Results:**

In HT-29/B6 cells, Uzara inhibited forskolin- as well as cholera toxin-induced I_SC_ within 60 minutes indicating reduced active chloride secretion. Similar results were obtained in human colonic biopsies pre-stimulated with forskolin. In HT-29/B6, the effect of Uzara on the forskolin-induced I_SC_ was time- and dose-dependent. Analyses of the cellular mechanisms of this Uzara effect revealed inhibition of the Na^+^/K^+^-ATPase, a decrease in forskolin-induced cAMP production and a decrease in paracellular resistance. Tracer flux experiments indicate that the dominant effect is the inhibition of the Na^+^/K^+^-ATPase.

**Conclusion and Implications:**

Uzara exerts anti-diarrheal effects via inhibition of active chloride secretion. This inhibition is mainly due to an inhibition of the Na^+^/K^+^-ATPase and to a lesser extent to a decrease in intracellular cAMP responses and paracellular resistance. The results imply that Uzara is suitable for treating acute secretory diarrhea.

## Introduction

Diarrheal diseases have a major impact on morbidity and mortality worldwide. The subclass of secretory diarrhea is typically induced by bacterial pathogens like enterotoxic *E. coli* or, in the worst case, *Vibrio cholerae*. It is characterized by active secretion of chloride and/or bicarbonate into the intestine and subsequently by a loss of fluid [1]. Although oral rehydration therapy has reduced mortality during the past 30 years [2], the search for antisecretory agents is still of great importance.

Under physiological conditions, a moderate basal intestinal secretion of electrolytes and water maintains fluid homeostasis and provides the luminal solvent for digestive processes [1]. In quantity, electrogenic chloride secretion is the predominant anion secretory process. It is the result of the action of four membrane transporters acting in concert. First, chloride uptake across the basolateral membrane occurs by the Na^+^-K^+^-2Cl^−^ cotransporter (NKCC1). The Na^+^/K^+^-ATPase provides the driving force for this chloride uptake by discharging sodium across the basolateral membrane. Potassium is recycled via basolateral K^+^-channels, a process that maintains the lumen negative transepithelial potential. Finally, the apical chloride efflux is realized via cAMP-dependent low conductive Cl^−^ channels which are present in the apical membrane of secretory epithelial cells localized in crypt and surface epithelium [3], [4]. Water follows driven by osmotic forces via cell membrane aquaporin channels and via paracellular water channels formed by the tight junction protein claudin-2 [5].

To study epithelial secretory mechanisms, permanent intestinal epithelial cell lines like HT-29/B6 cells have been successfully used. These cells exhibit many of the features normally associated with a chloride secretory epithelium. The cAMP-dependent Cl^−^ secretion can e.g. be stimulated by forskolin or cholera toxin. Subsequently, a lumen-negative voltage appears and a short-circuit current (I_SC_) compatible with anion secretion can be measured [6], [7]. This experimental condition is ideal for analyzing antisecretory effects of compounds such as the Uzara root extract, using an Ussing chamber set-up for *in vitro* measurements.

Uzara originates from the root of the South African plant *Xysmalobium undulatum* (family Asclepiadaceae) which is also known as wild cotton, milk bush or bitterhout. As reviewed by van Wyk [8], *X. undulatum* has been used internally and externally, as decoction or as root powder, in traditional African medicine. Treated symptoms include stomach cramps, diarrhea, afterbirth cramps, and headache, but also wounds and abscesses. In Germany, Uzara was introduced into the pharmaceutical market in 1911. Here, it is usually used as alcoholic-aquaeus root extract to treat acute diarrhea [9]. Uzarin, xysmalorin, and their isomers allouzarin and alloxysmalorin have been identified as the major Uzara compounds [9]. All four compounds are cardenolide glycosides which exhibit similarities to those of *Digitalis purpura*. Thus, at high doses Uzara has digitalis-like effects on the heart [10]. Since pioneering in-vivo and in-vitro studies in the 1930s, the major effect of Uzara in the treatment of diarrhea and abdominal cramps has been ascribed to its influence on intestinal motor function by blocking smooth muscle cell depolarization through local stimulation of sympathetic neurons and thus diminishing peristalsis [11]–[13]. Other actions of Uzara are reviewed by van Wyk [8] and include antidepressant-like effects, possibly due to serotonin re-uptake inhibition, fibroblast growth stimulation, and antibacterial, antifungal, antiplasmodial and antioxidant activity.

Although Uzara has been used to treat diarrhea in Europe for 100 years, the underlying mechanisms are not fully understood. Therefore, in the present study the effects of Uzara on secretagogue-stimulated secretion were investigated in HT-29/B6 cells and in human colonic biopsies. Time- and dose-dependent inhibitory effects of Uzara on electrogenic Cl^−^ secretion are presented. Analyses of tracer fluxes and para- and transcellular resistance revealed that Uzara blocked forskolin-induced chloride secretion through a shift of trans- and paracellular ion movement from secretion towards absorption. As underlying mechanisms an inhibition of the sodium potassium pump and of cAMP synthesis could be identified.

## Methods

### Ethics statement

The colon biopsy protocol was approved by the local ethics committee of the Charité Berlin (EA4/098/09). Patients underwent diagnostic colonoscopy and had given their informed consent to taking additional samples.

### Cell culture

Monolayers of the human colon carcinoma cell line HT-29/B6 [7] were grown in 25 cm^2^ culture flasks containing RPMI 1640 medium (PAA laboratories, Cölbe, Germany) supplemented with 10% (v/v) fetal bovine serum (Biochrom, Berlin, Germany), 0.1 mg/ml streptomycin and 100 U/ml penicillin (PAA laboratories). Cells were cultured at 37°C in a humidified 5% CO_2_ atmosphere and splitted weekly. For experiments, cells were seeded either in 12 mm or in 30 mm cell filter supports (PCF 3.0 µm pore size; Ø 12 mm, 8⋅10^5^ cells/filter or Ø 30 mm, 5⋅10^6^ cells/filter; Millipore, Eschborn, Germany) and cultured for 7–10 days to confluence.

### Substance applications

Uzara (Uzara® Solution N, Stada, Bad Vilbel, Germany) was purchased as dry extract with a concentration of 40 mg/ml glycosides in a alcoholic solution (43%). Forskolin and nystatin (Sigma-Aldrich, Steinbach, Germany) were dissolved in dimethylsulfoxid (DMSO). Ouabain (Merck, Darmstadt, Germany), fluorescein and cholera toxin (Sigma-Aldrich) were dissolved in water. Stock solutions were further dissolved with bathing medium to achieve final DMSO or ethanol concentration below 0.1%. All other chemicals, unless otherwise noted, were purchased from Sigma-Aldrich.

### Electrophysiology

For electrophysiological measurements, confluent cell layers on filter supports (Millicell PCF 3.0 µm pore size, Ø 12 mm, effective area 0.6 cm^2^) were directly mounted in conventional Ussing chambers to measure the transepithelial resistance (R^t^ or TER, Ω⋅cm^2^; modified method described by Kreusel *et al.*, 1991). Standard bathing solution contained in mM: 140 Na^+^, 123.8 Cl^−^, 5.4 K^+^, 1.2 Ca^2+^, 1.2 Mg^2+^, 2.4 HPO_4_^2−^, 0.6 H_2_PO^4−^, 21 HCO_3_^−^, 10 D-(+)-glucose. Solution was constantly equilibrated with 95% O_2_ and 5% CO_2_, to keep a pH value of 7.4 at 37°C. In HCO_3_^−^ free solutions, NaHCO_3_ was replaced by equimolar concentrations of NaCl and 10 mM HEPES was added. pH was adjusted to 7.4 with NaOH and the solution was constantly equilibrated with 100% O_2_. Baseline values were measured prior to each experiment which includes the resistance of the bathing solution and the empty filter support.

Experiments were performed under short-circuit conditions. For uni-directional ion flux measurements 2.4 kBq/ml ^22^Na^+^ and 9.2 kBq/ml ^36^Cl^−^ (NEN Life Science Products, USA) were used and added either to the mucosal or serosal side (ms or sm direction, respectively). Three 20 min flux periods were analyzed. A 100 µl sample was taken from the application side upon initiation and mixed with 900 µl bathing solution. Samples (1 ml) were collected from the receiving side. Volumes were replaced with fresh solution. Radioactivity of ^22^Na^+^ was counted in a 1480 Wizard TM3 counter (Wallac, Rodgau-Jügesheim, Germany). Subsequently the samples were mixed with 4 ml Ultima Gold high flash-point liquid scintillation cocktail (Packard Bioscience, Groningen, Netherlands) and counted again for ^36^Cl^−^ in a Tri-Carb 2100TR Liquid Scintillation counter (Perkin Elmer, Boston, USA).

To asses cell viability in the absence and presence of Uzara over a period of 48 hours, transepithelial resistance measurements were carried out on filter supports in Petri dishes, using chop-stick type electrodes (STX-2, World Precision Instruments, Berlin, Germany) as previously described [14].

Biopsy specimens from distal colon (30 cm *ab ano*) were obtained endoscopically from three control patients (2 male and 1 female; range 39–66 years) undergoing colonoscopy for tumor exclusion who did not show inflammation, either macroscopically or microscopically. Tissues were transported to the laboratory on ice in oxygenated bath solution. Specimens were spread out under a dissection microscope and a plastic ring with an inner diameter of 2.5 mm was glued to the serosal side of the biopsy using histoacryl tissue glue (B. Braun, Melsungen, Germany). Subsequently, this ring was placed in a micro-container tightened with silicon rubber seals and mounted in specialized Ussing-type chambers as previously described [15], [16]. The time between taking the biopsies and mounting the tissues into Ussing chambers (0.049 cm^2^) was approximately 10–15 minutes. To avoid bacterial growth, piperacillin (50 mg/l) and imipenem (10 mg/l) were added to the bath solutions. Both antibiotics had no effect on I_SC_ at the concentration used.

### cAMP enzyme-linked immunoabsorbent assay (EIA)

Cell cultures growing on filter supports were incubated serosally with 50 µg/ml Uzara 30 minutes prior to serosal addition of 10 µM forskolin. Forskolin and Uzara alone were also tested and compared with untreated monolayers. 1 hour after forskolin addition, cells were washed twice with phosphate-buffered saline (PBS, PAA laboratories) and incubated with 0.1 M HCl for 20 minutes. Lysed cells were scraped off and centrifuged at 1000 ×g for 10 minutes. The protein content of the supernatant was determined by employing BCA protein assay reagents (Pierce, Rockford, Ill., USA) and using a plate reader (Tecan, Crailsheim, Germany). Samples were dissolved and applied in the cAMP EIA Kit according to the manufacturer's instruction (Cayman Chemicals, IBL, Hamburg, Germany). The reaction was measured at a wavelength of 420 nm using a Spectra Max 340 PC (Molecular Devices, Union City, California, USA).

### Na^+^/K^+^-ATPase activity

Determination of Na^+^/K^+^-ATPase activity in membrane fractions of HT-29/B6 cells was performed with minor modification according to Sadrzadeh *et al.* [17]. After washing with PBS, cells were scraped from the permeable supports or culture flasks and homogenized in imidazole buffer (20 mM, pH 7.4) containing EDTA (0.5 mM). Samples were homogenized by shearing repeatedly through a hypodermic needle with a diameter of 0.9 mm and then by another one measuring 0.45 mm (B. Braun). The membrane fraction was obtained by 5 min centrifugation at 200 g (4°C) and a subsequent centrifugation of the resulting supernatant at 43,000×g for 30 minutes (4°C). Pellets were re-suspended in histidine-imidazole buffer (40:40 mM, pH 7.1). The protein content was determined by the BCA protein assay reagents.

Assays were performed in microtiter plates measuring the formation of inorganic phophate (P_i_). P_i_ released from ATP by the ATPase activity was quantified from a color reaction by means of the Spectra Max 340 PC at a wavelength of 810 nm using a P_i_ concentration standard. The activity of the Na^+^/K^+^-ATPase (in nmol/(min·mg)) was calculated from the difference of the entire ATPase activity and the blocking properties of 2.5 mM ouabain, a specific inhibitor of the Na^+^/K^+^-ATPase (Merck). The effect of Uzara on the Na^+^/K^+^-ATPase was measured either by incubation of the cell monolayer with Uzara or by incubation of the membrane fractions directly with Uzara in the incubation medium. Each sample was determined in triplicates.

### Two-path impedance spectroscopy

Confluent cell layers on filter supports were directly mounted in chambers specially designed for impedance measurements. Paracellular (R^para^) and transcellular (R^trans^) were measured by two-path impedance spectroscopy as described in detail by Krug *et al.* [18]. Both hemichambers were filled with 10 ml circulating bath solution (composition equal to the solution used in the conventional Ussing chamber). A programmable frequency response analyzer (402, Beran Instruments, Devon, UK) in combination with an electrochemical interface (1286, Solartron Schlumberger, Farnborough, UK) was employed for application of sinusoidal currents in frequencies from 1 Hz to 65 kHz. R^trans^ and R^para^ are calculated from impedance spectra and fluxes of the paracellular marker, fluorescein (332 Da), which were obtained before and after chelating extracellular Ca^2+^ with EGTA.

### Statistical analysis

Data are expressed as means ± standard error of the mean (s.e.m.). Statistical analysis comparing two groups was performed by using Student's t-test. ANOVA with Bonferoni-Holm adjustment was used in cases of multiple comparisons. P<0.05 was considered significant.

## Results

### Effect of Uzara on the forskolin-induced increase in I_SC_ in HT-29/B6 cells

**Figure 1:**
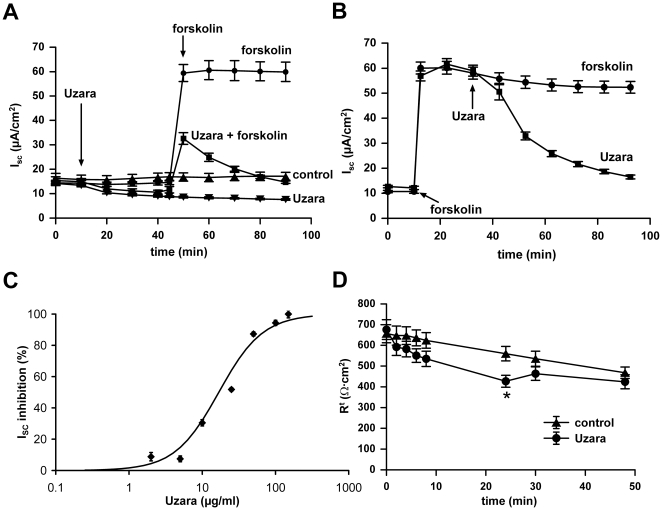
Inhibitory effect of Uzara on the forskolin-induced I_SC_ rise in HT-29/B6 cells. I_SC_ was measured on HT-29/B6 monolayers mounted in Ussing chambers. In A and B, Uzara (50 µg/ml) was added on the basolateral side either 30 minutes prior (A) or 30 minutes after (B) basolateral addition of 10 µM forskolin. In C, different Uzara concentrations were applied basolaterally 30 minutes after forskolin stimulation. The percentage of the inhibitory activity of Uzara to the forskolin-induced I_SC_ rise was calculated after an incubation time of 1 hour. An IC_50_ of 17 µg/ml and a Hill coefficient of 1.45 were determined from a Hill plot diagram and used to calculate the continuous line. All values are expressed as mean ± s.e.m. (n = 5-7). D: Transepithelial resistance measurements over 48 hours after application of either ethanol (control: final concentration 0.05%) or 50 µg/ml Uzara (final ethanol concentration 0.054%). With the exception of the resistances 24 hours after the application of uzara, none of the resistance values in the presence of uzara differed significantly from the corresponding control value. (means ± s.e.m. n = 12).

Monolayers of HT-29/B6 cells were mounted into Ussing chambers. Under resting conditions I_SC_ stabilized on a level of 17.1±1.6 µA/cm^2^ (n = 5). Uzara (50 µg/ml) added to the basolateral compartment slightly reduced I_SC_ to 7.6±0.3 µA/cm^2^ (n = 8) after 80 minutes of incubation (P<0.001). When forskolin (10 µM, basolaterally) was applied, I_SC_ rapidly increased to 59.9±4.0 µA/cm^2^ equivalent to a net transport of monovalent ions of 2.23±0.15 µmol/(h·cm^2^) (n = 6) and remained on this level for at least 80 min (Fig. 1A, P<0.001).

Two different experimental designs were used to characterize the anti-diarrheal effect of Uzara: (1) basolateral pre-incubation with Uzara (50 µg/ml) and subsequent addition of a secretagogue and (2) basolateral addition of Uzara after pre-stimulation of electrogenic transport by 10 µM forskolin for 30 minutes. Both experimental protocols yielded direct evidence for an antisecretory effect of Uzara (Fig. 1A and B). In contrast to basolateral addition, apical application of 50 or 100 µg/ml Uzara had no inhibitory effect on a forskolin-mediated I_SC_ increase (data not shown). Thus, the efficacy of Uzara was clearly restricted to basolateral application.

In addition, different Uzara concentrations (from 2 to 150 µg/ml) were tested for blocking the forskolin-stimulated I_SC_ response. Here, Uzara was applied to the basolateral compartment 30 minutes after addition of forskolin (Fig. 1C). The inhibitory effect of Uzara on the forskolin-induced I_SC_ increase was concentration-dependent with an IC_50_ of 17 µg/ml (estimated from a linear regression of values between 2 and 100 µg/ml in a Hill plot, n = 5-7, R^2^ = 0.93) and a Hill coefficient of 1.45.

All further experiments were carried out at Uzara concentrations of 50 µg/ml. To asses cell viability at this concentration, transepithelial resistance was measured over a time period of 48 h. As shown in Fig. 1D, transepithelial resistance between control cell layers (treated with ethanol only, final concentration 0.05%) and cell layers treated with Uzara did not differ significanly during the first 8 h after application. 24 h after application, Uzara-treated cell layers exhibited a reduced resistance, which, however, recovered over the following 24 h, so that 48 h after application there were again no significant difference between contol- and Uzara-treated cell layers. Therefore, even an exposure for 48 h to 50 µg/ml uzara did not affect cell viability. During all further experiments exposure to Uzara never exceeded 3 h.

### Effect of Uzara on the forskolin-stimulated cAMP level in HT-29/B6 cells

Forskolin increases the intracellular concentration of cAMP by activating the adenylate cyclase. Subsequently, this second messenger activates apical Cl^−^ channels required for the Cl^−^ efflux across the apical enterocyte membrane.

**Figure 2:**
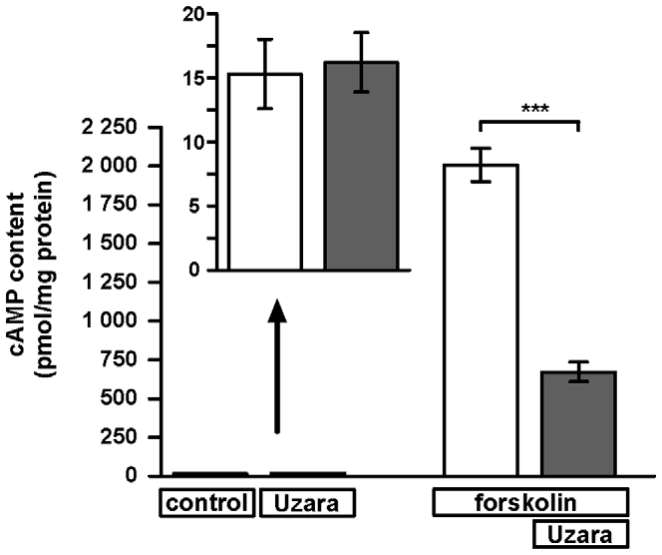
Inhibitory effect of Uzara on the forskolin-induced accumulation of intracellular cAMP in HT-29/B6 cells. HT-29/B6 monolayers were incubated basolaterally with 50 µg/ml Uzara 30 min prior to basolateral forskolin (10 µM) addition. After an incubation time of 1 h, cell lysates were prepared and the content of cAMP was assayed with EIA. The data shows that forskolin increased the intracellular cAMP level, whereas Uzara partly blocked this rise in cAMP. Values are expressed as mean ± s.e.m. (n = 5-6). Asterisks indicate difference versus control: ***P<0.001.

For testing the effect of Uzara on intracellular cAMP levels, cellular cAMP content was measured under the influence of forskolin and Uzara. HT-29/B6 cells growing on filter supports were pre-incubated for 30 minutes with Uzara before forskolin was added. 60 minutes after addition of forskolin cells were lysed for measuring the intracellular cAMP content. Forskolin increased the cAMP content up to 130-fold when compared to control and Uzara partially blocked this cAMP increase by almost 70% (Fig. 2, n = 5-6).

### Effect of Uzara on the activity of the Na^+^/K^+^-ATPase in HT-29/B6 cells

**Figure 3:**
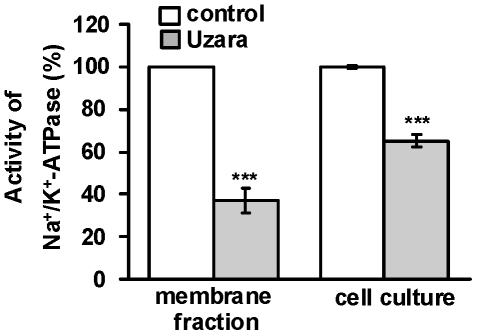
Inhibitory effect of Uzara on the Na^+^/K^+^-ATPase enzymatic activity in membrane preparations from HT-29/B6 cells. In cell culture experiments, monolayers were treated with 50 µ µg/ml Uzara for 90 min. Then, cells were homogenized and a crude membrane fraction was prepared. Herein, the Na^+^/K^+^-ATPase activity was measured (n = 5). In a further set of experiments, membrane fractions of untreated HT-29/B6 cell monolayers were prepared and subsequently treated with 50 µg/ml Uzara which was added for 1 h to the enzyme activity assay (n = 8). Both conditions revealed a direct inhibitory activity on the ion pump. Values are expressed as percentage of control activity. Basal activity of the Na^+^/K^+^-ATPase was between 36 and 73 nmol P_i_/(min·mg protein). Data are expressed as mean ± s.e.m. Asterisks indicate difference versus control: ***P<0.001.

The Na^+^/K^+^-ATPase participates in the process of electrogenic chloride secretion. For analyzing the Uzara effect on the activity of the Na^+^/K^+^-pump, the activity was measured in two types of experiments. First, HT-29/B6 cells grown on culture plate inserts were incubated basolaterally with 50 µg/ml Uzara for 1.5 hours. Subsequently, extracts of membrane fraction were prepared and tested (n = 8). Secondly, Uzara was added directly to the incubation medium of the assay (n = 5). Both incubation procedures clearly revealed an inhibitory activity of Uzara on the Na^+^/K^+^-ATPase. The inhibitory efficacy was higher when Uzara was directly applied to the assay than when added to the incubation medium of the cell monolayers (65±3% versus 37±6% inhibition of control values, respectively; Fig. 3).

### Effects of Uzara and ouabain on carbachol- and nystatin-induced transport in HT-29/B6 cells

**Figure 4:**
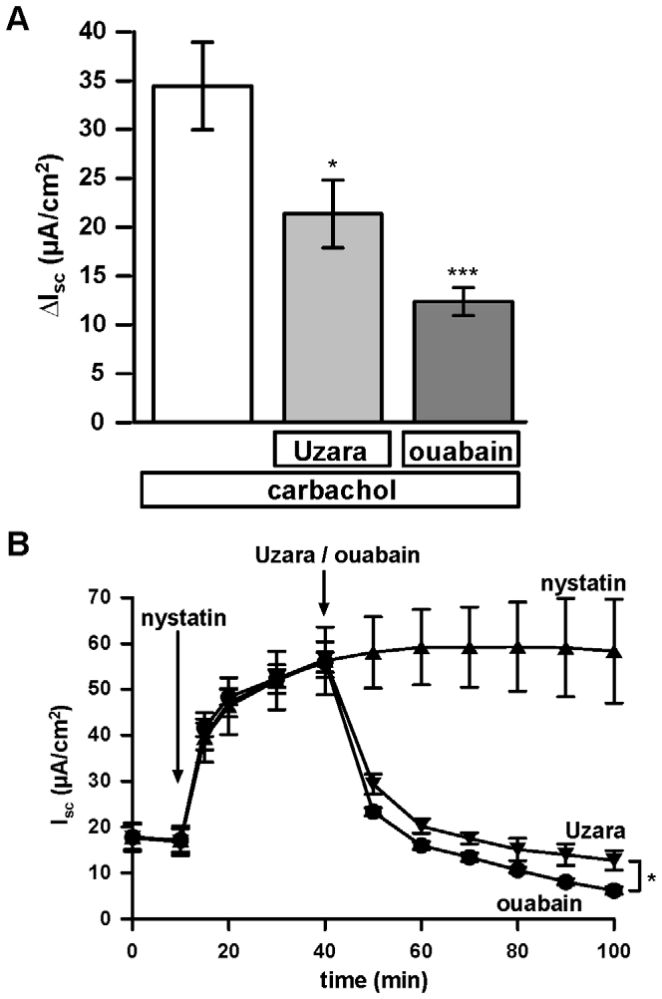
Inhibitory effect of Uzara and ouabain on the nystatin-induced I_SC_ rise in HT-29/B6 cells. The effects of Uzara and ouabain, a specific inhibitor of the Na^+^/K^+^-ATPase, were analyzed on the rise in I_SC_ induced by nystatin. Nystatin (15 µg/ml) which perforates cell membranes was applied to the mucosal compartment 30 min prior to basolateral addition of 50 µg/ml Uzara or 100 µM ouabain. I_SC_ was measured in HT-29/B6 cell monolayers mounted in Ussing chambers. The nystatin-induced rise in I_SC_ was diminished by Uzara as well as by ouabain. Data are expressed as mean ± s.e.m. (n = 4).

To further elucidate the mechanisms behind the effects of Uzara (50 µg/ml), ouabain (100 µM), carbachol (100 µM) and nystatin (15 µg/ml), respectively, were applied to HT-29/B6 cell layers. Carbachol induces an increase in the intracellular Ca^2+^ concentration and thereby activates basolateral, Ca^2+^-sensitive K^+^ channels and probably to a lesser extent also apical Ca^2+^-sensitive Cl^−^ channels. In the absence of a concomittant activation of apical, cAMP-sensitive Cl^−^ channels, this causes a brief, transient Cl^−^ secretion of about 2 min duration and a maximum amplitude of 34.4±4.5 µA/cm^2^. If cell layers were preincubated with Uzara or ouabain 30 min prior to carbachol application this amplitude was reduced to 21.3±3.5 and 12.3±1.4 µ µA/cm^2^, respectively (n = 6, Fig. 4A).

In contrast, apical application of nystatin, which is known to perforate cell membranes, increased I_SC_ reaching a plateau of about 54 µA/cm^2^. Addition of Uzara or ouabain after 30 min inhibited the nystatin-mediated I_SC_ rise. Ouabain was more effective than Uzara, in accordance with its stronger inhibitory effect on the Na^+^/K^+^-ATPase (Fig. 4B).

### Effect of Uzara and forskolin on Na^+^ and Cl^−^ fluxes in HT-29/B6 cells

Results of Na^+^ and Cl^−^ flux measurements are shown in Table 1. The secretagogue forskolin (10 µM) increased both, J_Cl_^ms^ (the mucosal-to-serosal Cl^−^ flux) and J_Cl_^sm^ (the serosal-to-mucosal Cl^-^ flux) but J_Cl_^sm^ to a greater extent, as a result of which net Cl^-^ flux was markedly increased in secretory direction.

**Table 1:** Influence of Uzara and forskolin on Na^+^ and Cl^−^ fluxes in HT-29/B6 cells.

	J_Na_^ms^	J_Na_^sm^	J_Na_^net^	J_Cl_^ms^	J_Cl_^sm^	J_Cl_^net^	J_res_	I_SC_	R^t^
control (n = 7)	0.67±0.09	0.62±0.06	0.04±0.08	0.94±0.13	1.31±0.06	−0.37±0.10	0.11±0.09	0.52±0.05	702±53
Uzara (n = 5)	0.72±0.23	0.50±0.03	0.22±0.22	0.90±0.22	1.03±0.06	−0.13±0.17	−0.08±0.19	0.28±0.02*	735±65
forskolin (n = 7)	0.66±0.06	0.81±0.09	−0.16 ±0.07	2.48±0.09#	4.35±0.16#	−1.87± 0.19#	0.83±0.25	2.54±0.12#	306±10#
Uzara+forskolin (n = 6)	0.89±0.08	1.00±0.19	−0.11±0.19	4.70±0.23§	5.11±0.13§	−0.41±0.24§	0.48±0.29	0.78±0.04§	316±16
ouabain+forskolin (n = 2)				3.60±0.07	4.27±0.15				

J_Na_ and J_Cl_, unidirectional Na^+^ and Cl^−^ fluxes; ^ms^, from mucosal to serosal side; ^sm^, from serosal to mucosal side; all fluxes are expressed as µmol·h^−1^·cm^−2^.

J^net^, net fluxes (J^ms^-J^sm^).

J_res_, residual fluxes (I_SC_-J_Na_^net^+J_Cl_^net^, usually assumed to reflect bicarbonate secretion).

I_SC_, short-circuit current (for comparison expressed in µmol·h^−1^·cm^−2^ of monovalent cations).

R^t^, transepithelial resistance (Ω·cm^2^).

All values represent means ± SEM.

*, P versus control <0.05.

#, P versus control <0.001.

§, P versus forskolin alone <0.001.

If Uzara was applied 30 minutes prior to forskolin addition, both Cl^−^ fluxes were increased. However, J_Cl_^ms^ was almost doubled, while J_Cl_^sm^ was only slightly affected. Thus, net flux analysis revealed that Uzara stimulated the Cl^−^ absorption and modulated the secretory Cl^−^ response to forskolin resulting in net Cl^−^ transport values of untreated monolayers. Uzara alone had no effects on the Cl^−^ flux.

In contrast to Cl^−^ fluxes, Na^+^ fluxes were not affected by forskolin and/or Uzara. Residual flux (usually assumed to reflect bicarbonate secretion), which showed a tendency to be enhanced by forskolin, was not reversed by Uzara. Finally, the forskolin-induced reduction in R^t^ was not altered by Uzara co-incubation.

### Effect of DIDS on the forskolin-induced I_SC_ in the presence or absence of Uzara

**Figure 5:**
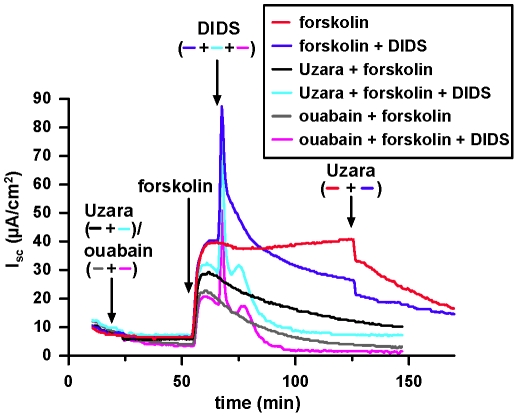
Effects of DIDS on forskolin-induced I_SC_ in the absence and presence of Uzara or ouabain. Experiment in HCO_3_^−^ free solutions equilibrated with 100% O_2_. Application of 10 µM forskolin induced an I_SC_ that was reduced in the presence of Uzara (50 µg/ml) and ouabain (100 µM). Under all three conditions, application of DIDS (100 µ µM) caused a pronounced transient increase in I_SC_, presumably due to an increase in intracellular Ca^2+^. Similar to the carbachol-induced I_SC_, the current amplitude was reduced in the presence of Uzara and ouabain. I_SC_ in the presence of DIDS then rapidly decreased, if preparations were pre-incubated with Uzara (light blue) or ouabain (pink). This effect is attributed to additional Na^+^ loading of the cells. Application of DIDS before the addition of Uzara (blue) had no such accelerating effect.

DIDS (0.1 mM) affected forskolin-induced I_SC_ only when applied to the basolateral side. The response consisted of a transient increase in I_SC_ followed by a decline below the plateau I_SC_ value prior to the addition of DIDS. These changes were not related to transport of bicarbonate, as they were still present in bicarbonate-free solutions equilibrated with 100% O_2_. Preincubation with Uzara did not affect the transient increase in I_SC_ but greatly accelerated the following decline in I_SC_ to basal values. An accelerated decline was also observed, if ouabain was used instead of Uzara, but was not observed if Uzara was added after the application of forskolin and DIDS (Fig. 5).

### Effect of Uzara or ouabain with or without forskolin on the para- and transcellular resistance in HT-29/B6 cells

**Figure 6:**
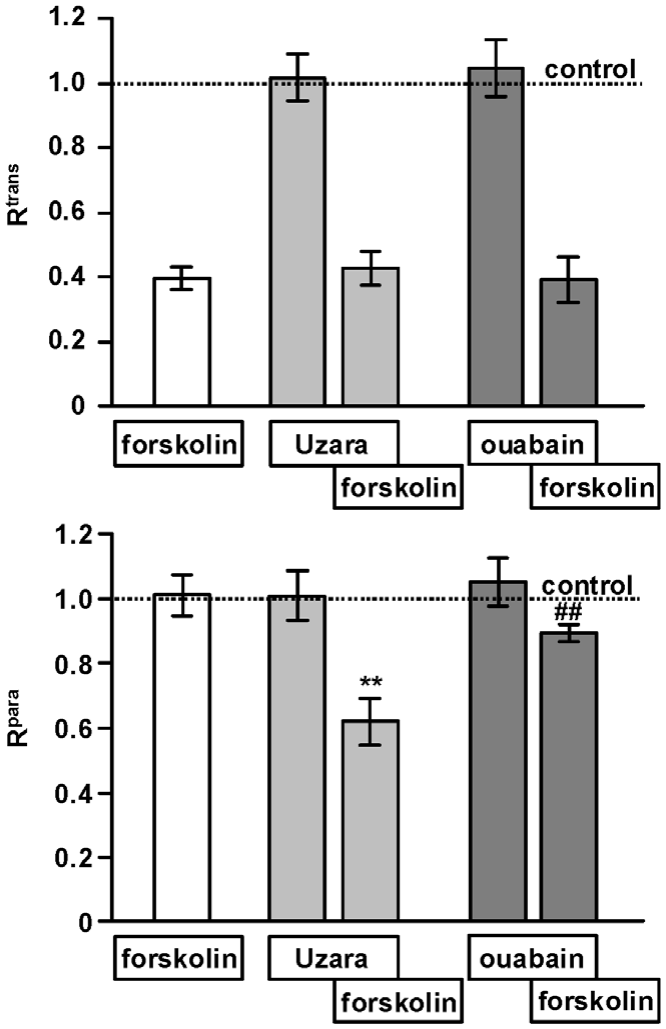
Effects of Uzara, ouabain and forskolin on R^para^ and R^trans^ in HT-29/B6 cells. The influence of Uzara and ouabain on R^para^ and R^trans^ with and without forskolin was determined by two-path impedance spectroscopy in HT-29/B6 cell monolayers mounted in Ussing chambers. Forskolin reduced R^trans^, whereas R^para^ remained unaltered. The co-incubation of Uzara with forskolin caused no change of R^trans^ compared to forskolin alone, whereas R^para^ was clearly reduced in the co-incubation group. Similarly, compared to forskolin alone, ouabain together with forskolin had no effect on R^trans^. The slight reduction of R^para^ observed under these conditions did not reach significance. Data are expressed as mean ± s.e.m. (n = 5-6). Untreated control values set as 1. Asterisk indicates a difference compared to forskolin: **P<0.01. Hash keys mean no statistical significance compared to forskolin alone.

The para- and transcellular resistance (R^para^ and R^trans^) were measured by two-path impedance spectroscopy, to decide whether the increase in J_Cl_^ms^ flux in the Uzara-forskolin group compared to forskolin alone was due to shifts in the paracellular or in the transcellular pathway. Results are shown in Fig. 6.

Uzara or ouabain alone had no influence on R^para^ and R^trans^. Forskolin mediated a reduction in R^trans^ of over 70% which was paralleled by Cl^−^ secretion and a R^t^ decrease. R^para^ remained unchanged by forskolin. The co-incubation of Uzara or ouabain with forskolin did not attenuate the forskolin-induced R^trans^ reduction. In contrast, R^para^ was significantly reduced by about 40% in the Uzara-forkolin group compared to forskolin. A slight R^para^ reduction observed in the presence of ouabain was not statistically significant.

### Effect of Uzara on cholera toxin-stimulated I_SC_ rise in HT-29/B6 cells

**Figure 7:**
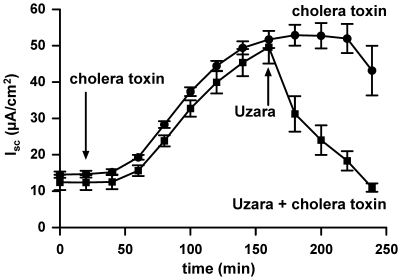
Inhibitory effect of Uzara on the cholera toxin-mediated I_SC_ rise in HT-29/B6 cells. I_SC_ was measured in HT-29/B6 cell monolayers mounted in Ussing chambers. Uzara (50 µg/ml) was applied on the basolateral side 150 min after mucosal addition of 1 µg/ml cholera toxin. The data show that Uzara diminished also the cholera toxin-induced rise in I_SC_. Values are expressed as mean ± s.e.m. (n = 6).

Effects of Uzara on the cholera toxin-induced I_SC_ rise was tested. Similar to forskolin, cholera toxin acts as an activator of the adenylate cyclase resulting in an increase of I_SC_ and Cl^−^ secretion. Mucosal addition of 1 µg/ml cholera toxin increased I_SC_ starting 40 minutes after addition and reaching a maximum after an incubation time of 150 minutes. At this time point, 50 µg/ml Uzara were applied basolaterally to the monolayer. As in the forskolin-induced design, Uzara also inhibited the cholera toxin-stimulated I_SC_ response reaching initial values again after 60 minutes (Fig. 7).

### Effect of Uzara on forskolin-stimulated I_SC_ rise in human colonic tissues

**Figure 8:**
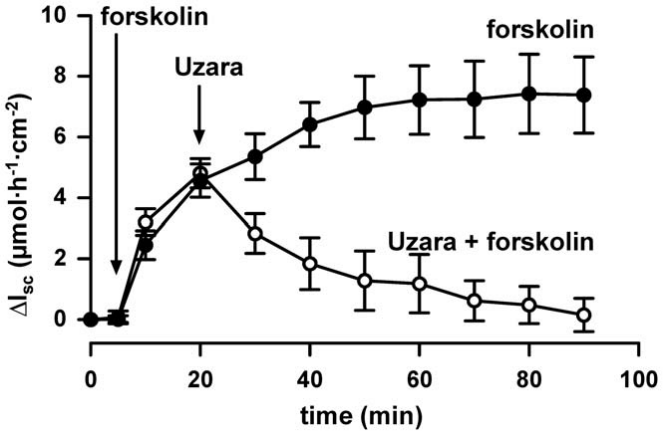
Inhibitory effect of Uzara on the forskolin-mediated I_SC_ rise in human colonbiopsy specimens. Delta I_SC_ (ΔI_SC_) was determined in human colon mounted in Ussing chambers. Forskolin (10 µM) was added to the basolateral side 15 min prior to basolateral addition of 50 µg/ml Uzara. Also in colonic biopsies, Uzara diminished the forskolin-induced rise in I_SC_. Data are expressed as mean ± s.e.m. (n = 5).

To test the Uzara effects not only on permanent intestinal cell cultures but also on native intestine, human colonic tissue specimens were investigated in the Ussing chamber. Tissues were pre-incubated basolaterally with forskolin, in order to induce an I_SC_ response. Then, Uzara was added basolaterally. As in HT-29/B6 cells, forskolin increased I_SC_ within minutes reaching a maximum after 40 minutes incubation. Uzara was applied 20 minutes after forskolin addition. Uzara diminished the forskolin-stimulated ΔI_SC_ reaching initial values after about 60 minutes (Fig. 8).

## Discussion

The plant Uzara has been used for a long time in traditional medicine to treat diarrheal disorders, but the mode of action is not fully known. In Germany, Uzara (Stada, Bad Vilbel, Germany) is frequently used as over-the-counter preparation of the Uzara root, containing 40 mg glycosides/ml. In our present study, the antisecretory effect of Uzara was investigated using colonic HT-29/B6 cell monolayers as well as native colonic tissue obtained during endoscopy.

Two different signaling pathways are involved in human colonic Cl^−^ secretion, a Ca^2+^-mediated and a cAMP-mediated secretory component [19], [20]. In both cases, Cl^−^ secretion is due to an activation of apical Cl^−^ channels and basolateral K^+^ channels. Ion movement is maintained by two basolateral ion transporters, NKCC1 and Na^+^/K^+^-ATPase [3], [4], [21].

To get more insight into the mechanisms of the antisecretory effect of Uzara, active intestinal secretion was stimulated with the secretagogues forskolin, cholera toxin and carbachol. Both, forskolin and cholera toxin lead to an activation of the andenylate cyclase and thus to an increase of intracellular cAMP. This induces a long-lasting electrogenic Cl^−^ secretion [22], [23] through co-activation of cAMP-sensitive Cl^−^ channels (CFTR; [3], [4]) and K^+^ channels (KCNQ1/KCNE3, [24]) in isolated intestinal preparations *in vitro* as well as in cultured cells of colonic origin. In contrast, carbachol, an agonist of muscarinic acetylcholine receptors, causes an increase in intracellular Ca^2+^ concentration and thus activates basolateral Ca^2+^-dependent K^+^ channels (KCNN4, [4]). At low cAMP levels, this only causes small increases in I_SC_, probably due to a scarcity in Ca^2+^-dependent Cl^−^ channels in the apical membrane. Increase in intracellular Ca^2+^ can, however, potentiate cAMP-induced Cl^−^ secretion [3], [25].

DIDS is commonly used as inhibitor of Cl^−^ channels or HCO_3_^−^-dependent carriers. However, in epithelial cells it has previously been observed that DIDS, similarly to carbachol, may stimulate Cl^−^ secretion by releasing Ca^2+^ from intracellular stores [26], [27].

In the present study, the Cl^−^ secretion induced by forskolin was measured as I_SC_ response in HT-29/B6. I_SC_ increase was apparent within seconds after forskolin addition, while the effect of cholera toxin had a delay of about 40 minutes and reached maximum values only after 2.5 hours. This delay in cholera toxin action is due to the complex chain of processes necessary to finally activate the adenylate cyclase and stimulate anion secretion [28]. The plant extract Uzara effectively diminished both, the secretory response to forskolin and to cholera toxin. Moreover, the inhibitory effect of Uzara could also be confirmed in human colon biopsies.

Uzara was found to have three major effects: a partial inhibition of the Na^+^/K^+^-ATPase, a partial inhibition of the forskolin-induced cAMP increase (inhibition of the adenylate cyclase and/or activation of the phosphodiesterase) and, in the presence of forskolin, a reduction in paracellular resistance. Of these, only inhibition of the Na^+^/K^+^-ATPase is shared with ouabain. Although not investigated in the present study, there are several reports that ouabain rather activates than inhibits the adenylate cyclase ([29], and references therein), and, as shown in Fig. 6, paracellular resistance is not affected by ouabain. Despite these differences, Uzara-induced inhibition of the Na^+^/K^+^-ATPase appeared to be the dominant effect in the present study, as the inhibitory effect of Uzara compared to ouabain was independent of the mode of Cl^−^ secretion stimulation, i.e. with (forskolin) and without (carbachol, nystatin) concomittant increase in intracellular cAMP levels. Furthermore, during submaximal forskolin stimulation, the inhibitory effect of Uzara relative to ouabain was not increased (data not shown), as would be expected, if reduced cAMP responses played a crucial role.

The increase in uni-directional Cl^−^ fluxes during the combined application of forskolin together with Uzara (and ouabain, respectively) is consistent with an increase in intracellular Na^+^ concentrations due to the inhibition of the Na^+^/K^+^-ATPase. Under these conditions, NKCC1 is close to its thermodynamic equilibrium and will therefore move ions across the basolateral membrane in both directions. Whereas Na^+^ and K^+^ permeabilities of the apical membrane in the presence of forskolin remain low, Cl^−^ can pass both membranes. Under conditions of NKCC1 equilibrium, this movement can therefore occur in both directions at equal rates. This interpretation is further supported by the results obtained during the application of DIDS (Fig. 5). Basolateral application of DIDS in the presence of forskolin caused an additional, transient Cl^−^ secretion that has been attributed to an intracellular Ca^2+^ release [26], [27] and that is also driven by NKCC1. During an inhibition of the Na^+^/K^+^-ATPase (in the presence of Uzara or ouabain) this accelerates loading of the cells with Na^+^, causing I_SC_ to rapidly approach baseline values again. Reversal of the experiment, i.e. application of Uzara after the application of DIDS, does not cause this rapid decrease in I_SC_, as DIDS-induced Na^+^ loading of the cell is prevented by the action of the Na^+^/K^+^-ATPase (Fig. 5).

Interestingly, control data from a small group point to a higher effect of Uzara than of ouabain on uni-directional Cl^−^ fluxes (respective ms- and sm-fluxes in µmol/(h·cm^2^): Uzara, 4.70±0.23 and 5.11±0.13, n  =  6 versus ouabain 3.60±0.07 and 4.27±0.15, n  =  2). This is most likely due to the Uzara-induced decrease in R^para^ that short-circuits the epithelial layer and thus prevents the maintenance of osmotically active gradients across the epithelium. This may further contribute to the anti-diarrheal effect of Uzara.

To date, several reports on extra-intestinal cells point to an inhibitory potential of Uzara glycosides on the Na^+^/K^+^-ATPase [10], [30], [31]. Uzara ingredients like uzarigenin and uzarin were shown to bind to the Na^+^/K^+^-ATPase and inhibit its activity. However, serum concentrations reached during treatment with Uzara remain below 10 ng/ml [32] and thus well below K_d_ values reported for the major Uzara compound, uzarin (∼700 Da), in the order of 1 µM (pig kidney ATPase[30]), 1.5 µM and 4.3 µM (cattle and guinea pig heart muscle ATPase, respectively [10]). This may explain, why Uzara administration in healthy subjects does not affect cardiovascular parameters evaluated by electrocardiography and impedance cardiography [33]. In contrast, at the site of Uzara absorption as result of an inflamed or attacked intestinal mucosa, local concentrations may well increase sufficiently to exert anti-secretory effects.

In conclusion, the traditional remedy Uzara exerts its anti-diarrheal effects not only through the previously reported inhibition of intestinal motility [11]–[13] but, in addition, through effects on the intestinal epithelium via inhibition of active secretion. This is caused by a partial inhibition of the Na^+^/K^+^-ATPase, by a reduction in intracellular cAMP responses (either through partial inhibition of the adenylate cyclase or by an activation of the phosphodiesterase), and by a decrease in paracellular resistance. It is inferred, that Uzara is suitable for treating secretory diarrhea caused e.g. by bacterial toxins as well as motility-related diarrhea, but may not be effective against chronic malabsorptive diarrhea with two exceptions. First, chologenic diarrhea is driven by deconjugated bile acid-induced anion secretion in the colon. Second, lipid malabsorption during steatorrhea leads to β-hydroxylation of fatty acids in the colon which can trigger active anion secretion. It is reasonable to assume that these two malabsorptive types of diarrhea are uzara-sensitive as well, although direct experimental evidence for both conditions is lacking.
